# Development of heart failure with preserved ejection fraction is independent of eosinophils in a preclinical model

**DOI:** 10.1002/iid3.1027

**Published:** 2023-09-27

**Authors:** Qi Pan, Cheng Chen, Zhaoting Gong, Guihao Chen, Yuejin Yang

**Affiliations:** ^1^ Department of Cardiology, State Key Laboratory of Cardiovascular Disease, Fuwai Hospital, National Center for Cardiovascular Diseases Chinese Academy of Medical Science and Peking Union Medical College Beijing China

**Keywords:** cardiac fibrosis, cardiac metabolism, diastolic function, eosinophils, heart failure with preserved ejection

## Abstract

The increasing burden of heart failure with preserved ejection fraction (HFpEF) has become a global health problem. HFpEF is characterized by systematic inflammation, cardiac metabolic remodeling, and fibrosis. Eosinophils act as an essential but generally overlooked subgroup of white blood cells, which participate in cardiac fibrosis, as reported in several recent studies. Herein, we explored the role of eosinophils in a “two‐hit” preclinical HFpEF model. The peripheral eosinophil counts were comparable between the normal chow and HFpEF mice. Deficiency of eosinophils failed to alter the phenotype of HFpEF. Conclusively, the development of HFpEF is independent of eosinophils in terms of the functional, biochemical, and histological results.

## Graphical abstract

This is the first report to evaluate the role of eosinophils in heart failure with preserved ejection fraction (HFpEF), where a novel preclinical model of HFpEF and eosinophil‐deficient mice was utilized. Results indicated that the development of HFpEF is independent of eosinophils, providing new insights into the roles of eosinophils in cardiovascular diseases.

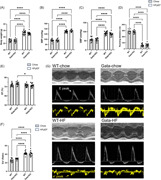

## 1 INTRODUCTION

With a rapid increase in its incidence and prevalence, heart failure (HF) is a major public health issue.^1^ Currently, HF with preserved left ventricular ejection fraction (HFpEF) has been recognized as the major form of HF, with a 5‐year survival rate after the first hospitalization of 35%–40%.^1^ HFpEF is a systemic syndrome characterized by diastolic dysfunction, classic HF symptomatology (shortness of breath, edema, and exercise intolerance), and a normal left ventricular ejection fraction (LVEF, >50%), involving heart, liver, skeleton muscle, adipose, and other tissue.^2^ Increasing evidence recognized the essential role of inflammation in the development of HFpEF, as demonstrated by elevated levels of plasma biomarkers (to name a few, C reactive proteins [CRP], tumor necrotic factor alpha [TNF‐α], and pentraxin 3 [PTX3]) and improved performance postanti‐inflammatory interventions.^2^

As an important but generally overlooked subgroup of white blood cells, eosinophil was considered as an antiparasitic and pro‐allergic player due to its capacity to produce galectin‐10, major basic protein (MBP), eosinophil cationic protein (ECP), eosinophil peroxidase (EPO), derivatives of arachidonic acid, and other active substances.^3^ Besides, eosinophils are able to present antigens to Th2 lymphocytes and activate dendritic cells by generating EPO.^4^ However, the role of eosinophil in cardiovascular diseases has been recognized recently. In a preclinical model of acute myocardial infarction, eosinophils facilitated the recovery of cardiac dysfunction due to CD206+ macrophage polarization via the interleukin (IL)‐4/STAT6 axis.^5^, ^6^ Despite the lack of direct investigations on heart failure with preserved ejection fraction (HFpEF), both mice and human eosinophil seemed to block Smad2 and Smad3 signaling in cardiac fibroblasts induced by transforming growth factor β (TGF‐β).^6^, ^7^

Given the above, eosinophils seem to be beneficial in HFpEF mice in theory. Herein, a novel preclinical model of HFpEF and eosinophil‐deficient mice was utilized to examine the association of eosinophils with HFpEF. However, we found no elevation of peripheral blood eosinophils in HFpEF mice. Mice with eosinophil deficiency showed no alternation of cardiac function and histological performance.

## 2 MATERIALS AND METHODS

### 2.1 Animal experiments

All animal experiments complied with the ARRIVE guidelines and were carried out in accordance with the UK Animals (Scientific Procedures) Act, 1986, and associated guidelines. All animal studies were approved by the Animal Care and Use Committee of Fuwai Hospital, Peking Union Medical College of Medicine (No. FW‐2022‐0041). A total of 50 male wild type (WT, 6‐week‐old, 18–22 g, Charles River) and 50 ΔdblGATA1 knock‐out (Gata1 KO, 6‐week‐old, 18–22 g, #033551; The Jackson Laboratory) C57BL/6N mice, which have complete ablation of the eosinophil lineage,^8^ were utilized to assess the effects of eosinophils on the development of HFpEF. All animals utilized in this study were kept in a specific pathogen‐free barrier system with free access to the standard laboratory chow diet and kept at a 12‐h light/dark cycle in cages (3–4 mice per cage). During the study, mice with rapid body weight loss, cachexia, or agonal stage were excluded from the analysis and euthanized by carbon dioxide anesthesia. At the end of the study, all mice were anesthetized with 2% tribromoethanol and killed.

Mice were randomly assigned into two groups with cage as the experimental unit, which were exposed to normal diet (chow) or high‐fat diet (HFD, #D12492, Research Diet) plus *N*[w]‐nitro‐l‐arginine methyl ester (l‐NAME, 0.5 g/L in drinking water with pH adjusted to 7.4, #S2446; Sigma Aldrich) for 12 weeks, respectively for establishment of HFpEF, as reported previously.^9^, ^10^, ^11^ Investigators were masked until the analysis was finished. Both locations of cages and all experiments were in random order to control confounders.

### 2.2 Peripheral blood cell count

A total of 150 μL peripheral blood was obtained from the eye socket. The blood samples were diluted to three times (total volume, 450 μL) by adding normal saline, and then evaluated by blood cell counter 2120i (Siemens).

### 2.3 Noninvasive blood pressure measurement

Both systolic blood pressure (SBP) and diastolic blood pressure (DBP) were measured noninvasively in conscious mice using the tail‐cuff instrument (BP‐2010; Softron). Animals were placed in individual holders on a temperature‐controlled platform (37°C), and recordings were performed under steady‐state conditions after sufficient acclimatization. The averaged values of three measurements of each mouse were included in the analysis.

### 2.4 Transthoracic echocardiography

Echocardiography was undertaken by employing the Visual Sonics Vevo 2100 imaging system (FUJIFILM). The mice were anesthetized with 3% isoflurane and maintained with 1%–2% isoflurane in 95% oxygen. Anesthesia was adjusted to obtain a target heart rate of 470 ± 50 beats per minute (bpm). Left ventricular (LV) ejection fraction (EF) was measured from the short‐axis M‐mode at the mid‐ventricular level. Apical four‐chamber view was used to obtain tissue Doppler imaging (TDI) mode and Pulse‐wave Doppler (PWD) mode for analysis of myocardial velocity (to obtain e' value) and blood flow velocity (to obtain E value), respectively. All parameters were measured three times, and means were used for analysis. At the end of the procedures, all mice recovered from anesthesia without difficulties.

### 2.5 Exhaustion test

Mice ran uphill (10°) on the FT‐200 treadmill (Techman) starting at a warm‐up speed of 4 m/min for 4 min after which speed was increased to 9 m/min for 2 min. The pace was then increased by 2 m/min per 2 min till mice were exhausted, which was defined as the inability of the mouse to return to running within 10 s of direct contact with an electric‐stimulus grid. Record running time and calculate running distance.

### 2.6 Histological evaluation and immunofluorescence staining

After anaesthetization, the hearts and bilateral lungs were harvested from the mice and weighed. The lungs were dried under 65°C for 3 days and weighed again. Length of tibias was measured by a vernier caliper. Then hearts were fixed in 4% PFA for 60 ± 6 h and imbedded in paraffin. Slides (5‐μm thick) were acquired, baked at 68°C for 45 min, and stained with hematoxylin and eosin (H&E) or Sirius Red solution by routine methods.^12^ Fibrosis rate was calculated as fibrotic area (red area in Sirius Red‐stained slides)/total LV area.

Wheat germ agglutinin (WGA, conjugated with Alexa Fluor 594, ThermoFisher) staining was used to examine cross cross‐sectional area of cardiomyocytes, an indicator of cardiac hypertrophy. For immunofluorescence staining, the paraffin sections were incubated with the antibodies at 4°C overnight, followed by goat antimouse (ab150116, 1:200 dilution; Abcam) or goat antirat (ab150165, 1:200; AbcamA) conjugated with Alex Fluro 488 or 594 secondary antibodies for 1 h at room temperature. After washing, the nuclei were stained with 4′,6‐diamidino‐2‐phenylindole. The sections were then observed under a laser scanning confocal microscope (Leica) at ×400 magnification and five randomly chosen high‐power fields (HPF) were measured per animal. Primary antibodies in the study included rat anti‐mouse Siglec‐F antibody (14‐1702‐82, 1:50; ThermoFisher) and mouse anti‐mouse cardiac Troponin T (cTnT, ab8295, 1:200; Abcam). For each mouse, five HPF images were obtained, and the averaged eosinophil count or cross‐sectional area was utilized for quantitative analysis.

### 2.7 Quantitative real‐time PCR and enzyme‐linked immunosorbent assay (ELISA)

Total RNA was extracted from heart tissue with Trizol reagent (Life Technologies) according to the manufacturer's instructions. Reverse transcription was performed using a Prime‐ScriptTMRT Reagent Kit with gDNA Eraser (Takara). The quantitative real‐time PCR process of Gapdh and Nppb (encoding B‐type natriuretic peptide [BNP]) was conducted with PowerUp™ SYBR™ Green Master Mix (Applied Biosystem) on a QuantStudio 3 Real‐Time PCR system (Applied Biosystem). The data were calculated via comparative 2^−ΔΔCt^ methods. The levels of serum transforming growth factor β (TGFβ) was measure using mouse TGFβ ELISA kit (#RK00057; Abclonal) according to the manufacturer's instructions.

### 2.8 Statistical analysis

Accurate *N* for each study were recorded in the figure legends. Statistical data are presented as the mean ±  standard deviation (SD) and were analyzed using GraphPad 8.0 (GraphPad Software). Outliers, which means higher than mean +2 SD or lower than mean −2 SD, would be excluded during analysis. Comparisons between two groups were analyzed by unpaired student *t*‐test. Comparisons among four groups were evaluated by two‐way analysis of variance. Statistical significance was set at *p* < .05 for all comparisons.

## 3 RESULTS

### 3.1 Peripheral eosinophil counts were comparable between normal chow and HFpEF mice

**Figure 1:**
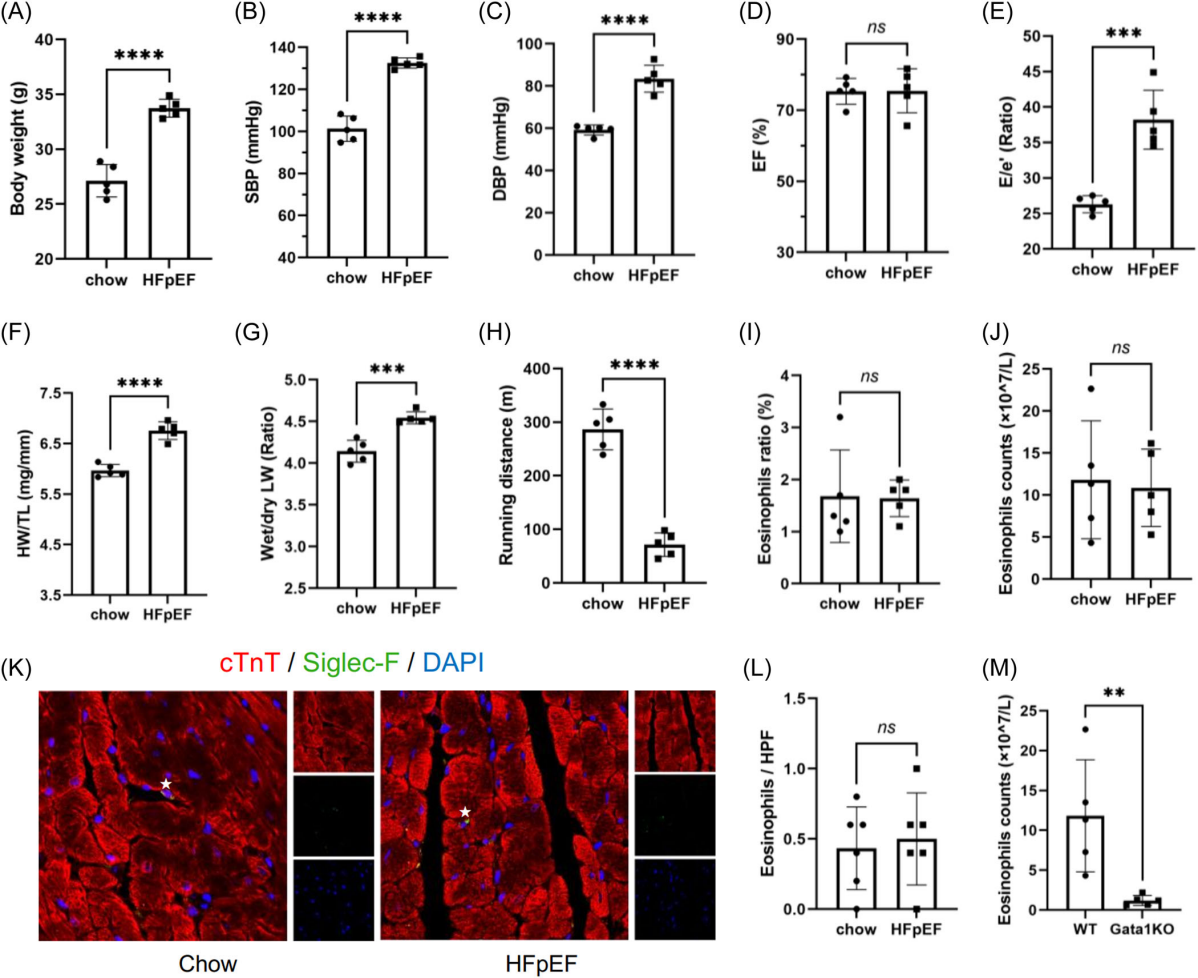
The development of cardiometabolic heart failure with preserved ejection fraction (HFpEF) model and the circulating eosinophil counts. (A) Body weight of the chow and HFpEF mice. (B) Systolic blood pressure (SBP) of the chow and HFpEF mice. (C) Diastolic blood pressure (DBP) of the chow and HFpEF mice. (D) Left ventricular ejection fraction (LVEF) of the chow and HFpEF mice. (E) the ratio of E to e' (E/e') of the chow and HFpEF mice. (F) The ratio of heart weight to tibial length (HW/TL) of the chow and HFpEF mice. (G) The ratio of wet lung weight (LW) to dry LW of the chow and HFpEF mice. (H) Running distance of the chow and HFpEF mice. (I) Proportion of eosinophils in peripheral white blood cells of the chow and HFpEF mice. (J) Absolute peripheral eosinophil counts of the chow and HFpEF mice. (K) Eosinophil (Siglec‐F +) infiltration in myocardium of chow and HFpEF mice (stars). (I) Quantificative analysis of averaged infiltrated eosinophils per HPF. (M) Comparison of circulating eosinophils between WT and Gata1 KO mice measured by peripheral blood cell counts. For all analyses, *n* = 5 for each group. *p < .05, **p < .01, ***p < .001, **** *p* < .0001. DAPI, 4′,6‐diamidino‐2‐phenylindole; ns, not significant.

As reported in the previous study,^10^ the HFpEF mice model induced by HFD and l‐NAME recapitulated a bulk of clinical features of patients of HFpEF, including obesity (Figure 1A), hypertension (Figure 1B,C), normal systolic function (Figure 1D), and impaired diastolic function (Figure 1E and Supporting Information: S1A). The combined treatment led to significant cardiac hypertrophy and pulmonary edema in terms of the elevation in the ratio of heart weight to tibial length (HW/TL; Figure 1F) as well as the ratio of wet lung weight (LW) to dry LW (Figure 1G), respectively. The exercise tolerance was also impaired in HFpEF mice (Figure 1H). Significant fibrosis and cardiomyocyte hypertrophy were observed in HFpEF mice (Supporting Information: Figure S1B–E). No significant difference in circulating eosinophil proportion and absolute counts as well as infiltrated numbers of eosinophils were observed between the chow group and HFpEF group (Figure 1I–L). To assess the effects of eosinophils on HFpEF development, we used Gata1 KO mice, whose eosinophils were depleted.^8^ As shown in Figure 1K, peripheral eosinophil counts decreased by 89.8% in Gata1 KO mice.

### 3.2 Eosinophil‐deficiency didn't alter functional phenotypes of HFpEF

**Figure 2:**
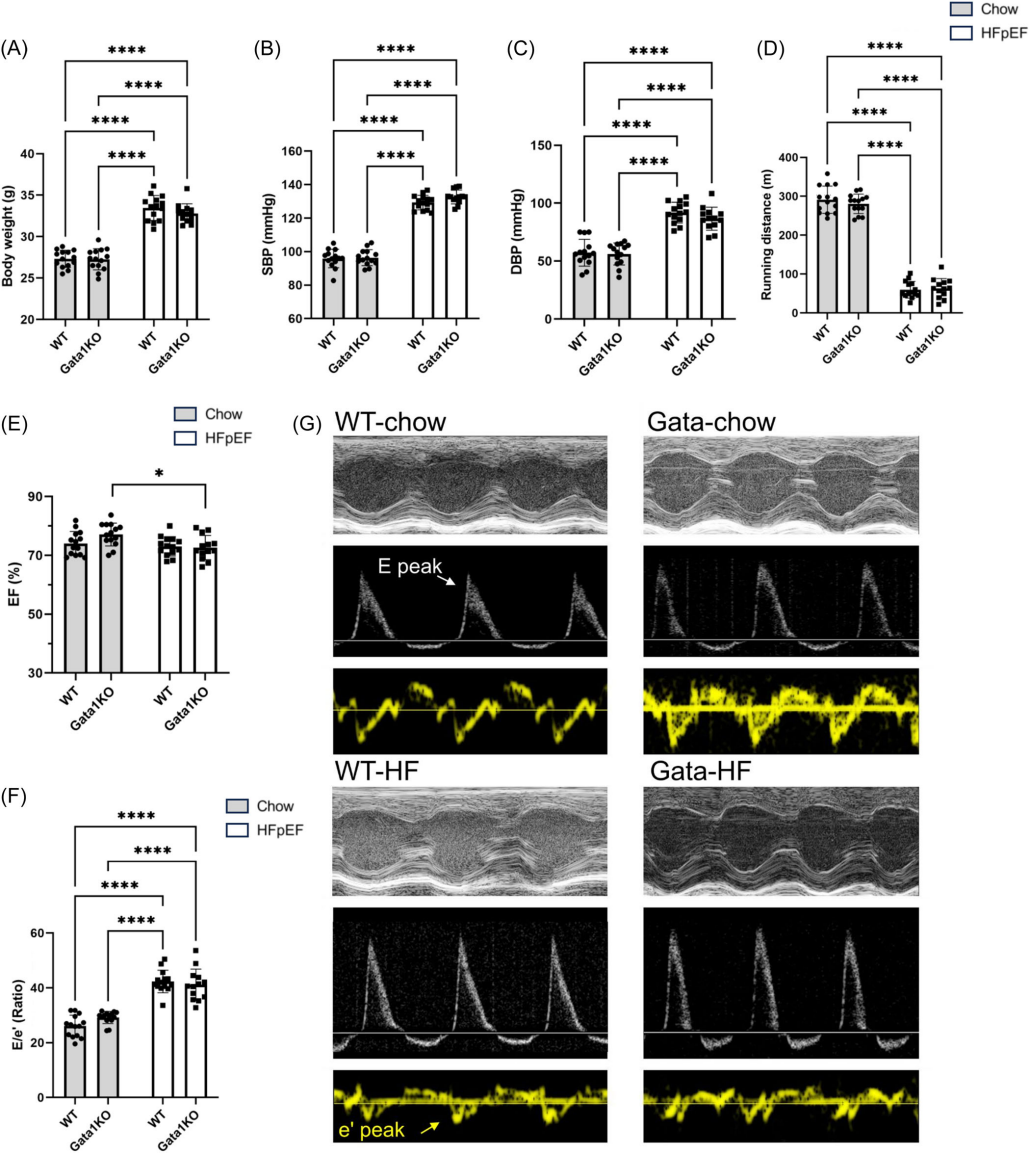
Evaluation of the effects of eosinophils on diastolic cardiac function in the HFpEF model. (A) Body weight of the mice in the WT‐chow, Gata‐chow, WT‐HFpEF, Gata‐HFpEF groups. (B) SBP of the mice in four groups. (C) DBP of the mice in four groups. (D) Running distance of the mice in four groups. (E and F) Quantitative analysis of LVEF (H) and E/e' ratio (I). (G) Representative images of echocardiography of the four groups, showing E peak and e' peak. For all analysis, *n* = 14, 14, 14, and 13 for the WT‐chow, Gata‐chow, WT‐HFpEF, and Gata‐HFpEF groups. *p < .05, **p < .01, ***p < .001, ****p < .0001. DBP, diastolic blood pressure; HFpEF, heart failure with preserved ejection fraction; ns not significant; SBP, systolic blood pressure; WT, wild type.

WT and Gata1 KO mice had comparable degrees of obesity (Figure 2A), SBP (Figure 2B), and DBP (Figure 2C). We didn't observe a significant difference in the degree of exercise performance impairment between WT‐HFpEF and Gata1‐HFpEF mice (Figure 2D). Echocardiography was utilized to assess the diastolic function of the heart in the groups (Figure 2E–G). LVEF were similar among different groups. Interestingly, a mild decline was observed between the Gata‐chow and Gata‐HFpEF groups (76.95% ± 3.87% vs. 71.90% ± 4.70%, *p* = .011), both of which were considered to be normal. Eosinophil‐deficient mice did not show significantly altered diastolic function, as demonstrated by the comparable ratios of E to e' in WT‐HFpEF and Gata‐HFpEF groups (Figure 2F).

### 3.3 Eosinophil‐deficiency didn't alter cardiac hypertrophy and fibrosis

**Figure 3:**
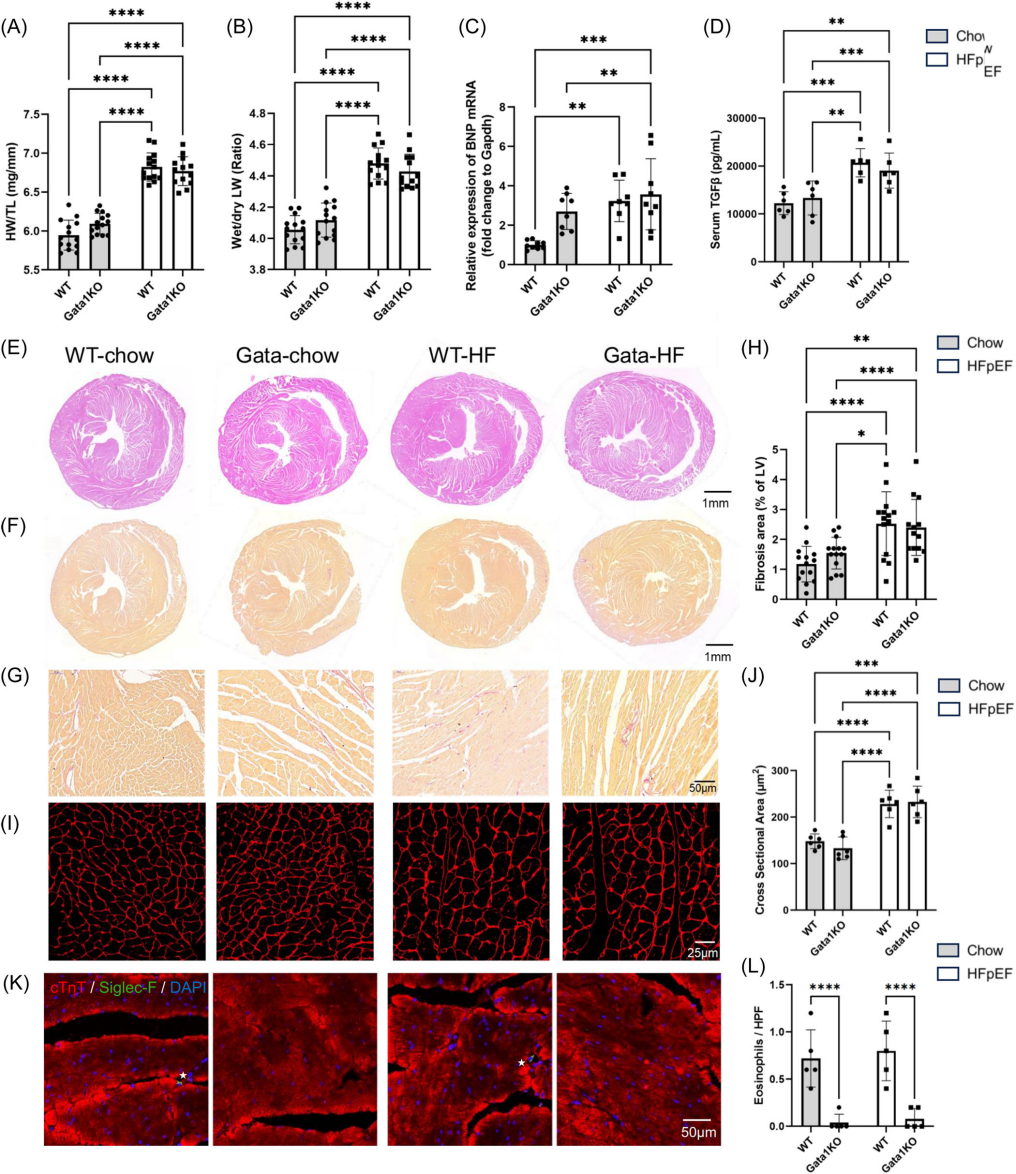
Evaluation of the effects of eosinophils on hypertrophy and fibrosis in the HFpEF model. (A) The HW/TL ratio in the WT‐chow, Gata‐chow, WT‐HFpEF, and Gata‐HFpEF groups. (B) The ratio of wet LW to dry LW in the four groups. (C) The relative expression of B‐type natriuretic peptide (BNP) mRNA measured by qRT‐PCR. (D) The serum TGFβ levels in the four groups. (E) H&E staining of heart tissue (low power field). (F and G) Sirius Red staining of heart tissue (F, low power field; G, high power field). (H) Quantitative analysis of fibrosis area. (I) Representative images of WGA staining. (J) Quantitative analysis of cross‐sectional area based on WGA staining. (K and L) Representative images and quantitative analysis of eosinophil (Siglec F+; stars) infiltrated in myocardium. For all analyses except WGA, immunofluorescence, qPCR, and ELISA, *n* = 14, 14, 14, and 13 for the WT‐chow, Gata‐chow, WT‐HFpEF, and Gata‐HFpEF group. For qRT‐PCR, *n* = 9, 8, 8, and 9, respectively. For WGA and ELISA, *n* = 6 for each group. For immunofluorescence staining, *n* = 5 for each group. *p < .05, **p < .01, ***p < .001, ****p < .0001. ELISA, enzyme‐linked immunosorbent assay; H&E, hematoxylin and eosin; HFpEF, heart failure with preserved ejection fraction; HW, heart weight; ns not significant; LW, lung weight; mRNA, messenger RNA; qRT‐PCR quantitative reverse‐transcription polymerase chain reaction; TGF‐transforming growth factor; TL, tibial length; WGA, wheat germ agglutinin; WT, wild type.

WT‐HFpEF and Gata‐HFpEF groups had similar ratios of both HW/TL (Figure 3A) and wet LW/dry LW (Figure 3B), indicating eosinophil removal had no effects on myocardial hypertrophy and pulmonary edema in HFpEF mice, respectively. The relative expression of BNP mRNA also displayed no difference between the WT‐HFpEF group and the Gata‐HFpEF group (Figure 3C). We also assessed the serum TGFβ levels, an essential inflammatory factor that promotes fibrosis. HFD and l‐NAME increased TGFβ levels in both WT and Gata1 KO mice (Figure 3D). However, the levels in WT‐HFpEF and Gata1‐HFpEF mice were comparable. For the histological section, H&E and Sirius Red staining were performed to assess fibrosis in the heart. No difference in the degree of hypertrophy and fibrotic rate was observed between the WT‐HFpEF group and the Gata‐HFpEF group, as shown by the Sirius Red staining (Figure 3E–H). WGA staining was performed to assess cardiac hypertrophy (Figure 3I,J), and the cross‐sectional areas were comparable between the two chow groups and between the two HFpEF groups. What's more, we noticed that there were no significant differences in the levels of blood eosinophils and infiltrated eosinophils in the myocardium between the two chow groups or between the two model groups (Figure 3K,L and Supporting Information: S2). Conclusively, all these results demonstrated eosinophil deficiency did not lead to improvement or deterioration of cardiometabolic HFpEF.

## 4 DISCUSSION

In this study, we assessed the effects of eosinophils in HFpEF. A novel, well‐recognized “two‐hit” model was applied, characterized by metabolic inflammation (induced by obesity) and hypertensive stress (induced by l‐NAME, which inhibits of constitutive nitric oxide synthase). This preclinical model is favored because it recapitulates the numerous systemic and cardiovascular features of HFpEF people, such as diastolic dysfunction, preserved LVEF, exercise intolerance, elevated LV filling pressures, microvascular dysfunction, modest cardiomyocyte hypertrophy, capillary rarefaction, predisposition to AF, and modest myocardial fibrosis.^10^ The level of circulating eosinophil didn't alter in HFpEF mice, and the development of HFpEF seemed to be eosinophil‐independent, as revealed by the eosinophil‐deficient mice. To the best of our knowledge, this is the first report to evaluate the role of eosinophils in HFpEF.

The role of inflammation has been of particular interest in recent investigations on heart failure, including HFpEF. Myocyte chemoattractant protein 1 (MCP‐1), an essential cytokine regulating the migration and infiltration of monocytes/macrophages, was significantly activated in response to pressure overload, and the inhibition of MCP‐1 alleviated diastolic dysfunction.^13^ IL‐16 was reported elevated in a rat preclinical model of HFpEF, which was positively associated with LV end‐diastolic pressure and LV stiffness constant.^14^ In another recent report, the levels of TNF‐α and IL‐1β and the infiltrated immunocytes were significantly higher in the “two‐hit” HFpEF model, leading to evoked pyroptosis in the epicardial adipose tissue/myocardium axis.^15^ Besides, blockage of IL‐17 receptor attenuated the progression of fibrosis and diastolic dysfunction in the hypertension‐related HFpEF rat model.^16^ Given the findings, discussion of the effects of specific immunocyte subpopulations in the development of HFpEF could contribute to understanding the pathogenesis and develop novel therapies.

Despite several advances recently, the role of eosinophils in myocardium remains to be elucidated due to inconsistent observations. For instance, higher eosinophil count was considered as a risk factor or predictor of several cardiovascular diseases. Cardiac syndrome X (CSX), characterized by exertional angina pectoris and normal coronary angiography results, may involve microvascular spasm and endothelial dysfunction. It's reported that patients with CSX had higher circulating eosinophil counts compared with control persons.^17^ Similarly, increased levels of peripheral eosinophils have been associated with the risk of coronary artery disease.^18^ In addition, Ong et al. claimed that the absence of natural killer (NK) cells led to the increased influx of eosinophils (up to 10 folds) and the resultant deterioration of fibrosis and myocarditis.^19^ In rare examples, excessive myocardial infiltration can result in eosinophilic myocarditis. The production of eosinophil‐specific active substances contributes to thrombotic complications and sometimes myocardial necrosis (eosinophil toxicity).^20^ Results in this study indicated that antieosinophil strategy seemed to be safe in terms of HFpEF. On the other hand, eosinophils are also recruited to the infarcted heart, and the increase of eosinophils to two‐fold seems to block hypoxia and reactive oxygen species‐induced cardiomyocyte apoptosis, promote CD206+ macrophage polarization and dampen fibroblast activation.^5^, ^6^, ^7^ These results demonstrated that the role of the eosinophils in the heart under different conditions might vary, and separate studies in the models are required. In theory, the absence of eosinophil may be detrimental because eosinophil seems to be an inhibitor of fibrosis and inflammation, which are also principal features of the “two‐hit” HFpEF model pathophysiology.^10^ However, eosinophils didn't increase in HFpEF mice in this study. Deficiency of eosinophils didn't lead to altered phenotypes of heart failure and myocardial hypertrophy in terms of blood pressure, echocardiographic performance, and histological assessments. This may be attributed to the double‐side effects of eosinophils on cardiomyocytes, which is like a seesaw. When it comes to HFpEF, a balance between eosinophil toxicity and the anti‐fibrosis and anti‐inflammation effects is reached.

There are some limitations to be noted. First, molecular mechanisms underlying the marginally insignificant upregulation of BNP in Gata1‐chow mice compared with WT‐chow mice were not explored. Additionally, we observed a trend of mild spontaneous cardiac hypertrophy and fibrosis in Gata1 KO mice in terms of E/e', HW/TL and Sirius Red staining. However, none of the indicators changed sharply, and most importantly, the running distance is comparable between the two groups, indicating the clinical significance of these changes is limited. Besides, it's infeasible to upregulate the eosinophils by IL‐5 to further verify the conclusion because administrating cytokines for a long time could be difficult due to high cost, and the adverse effects of IL‐5 such as pro‐anaphylaxis, body loss and global immune alternations result in failure of modeling and unreliable interpretation of results. Lastly, the roles of eosinophils in other HFpEF phenogroups (such as age‐related phenogroup and hypertension‐related phenogroup) were not discussed, although the “two‐hit” model is generally considered the best preclinical model of HFpEF to imitate clinical conditions of the majority of patients.^1^

Conclusively, the present study revealed that eosinophils did not drive the development of cardiometabolic HFpEF. However, more investigations are required to explore the mechanisms of mild spontaneous cardiac hypertrophy and fibrosis caused by eosinophil deficiency.

## AUTHOR CONTRIBUTIONS

**Qi Pan**: Conceptualization; formal analysis; investigation; methodology; software; validation; and writing—original draft. **Cheng Chen**: Conceptualization; formal analysis; methodology; software; and validation. **Zhaoting Gong**: Data curation and resources. **Guihao Chen**: Funding acquisition; project administration; supervision; visualization; and writing—review & editing. **Yuejin Yang**: Data curation; funding acquisition; project administration; resources; supervision; and writing—review & editing.

## CONFLICT OF INTEREST STATEMENT

The authors declare no conflict of interest.

## ETHICS STATEMENT

All animal studies were approved by the Animal Care and Use Committee of Fuwai Hospital, Peking Union Medical College of Medicine (No. FW‐2022‐0041).

## Supporting information

Supporting information.

## Data Availability

The data that support the findings of this study are available from the corresponding author upon reasonable request.
