# Supplementary material for: Development of heart failure with preserved ejection fraction is independent of eosinophils in a preclinical model
Source: Immun Inflamm Dis. 2023 Sep 27;11(9):e1027. doi: 10.1002/iid3.1027 (PMC10523958; doi:10.1002/iid3.1027)
Supplement: Supplementary file 1 — Supporting information. [file IID3-11-e1027-s001.docx]

Supplementary materials


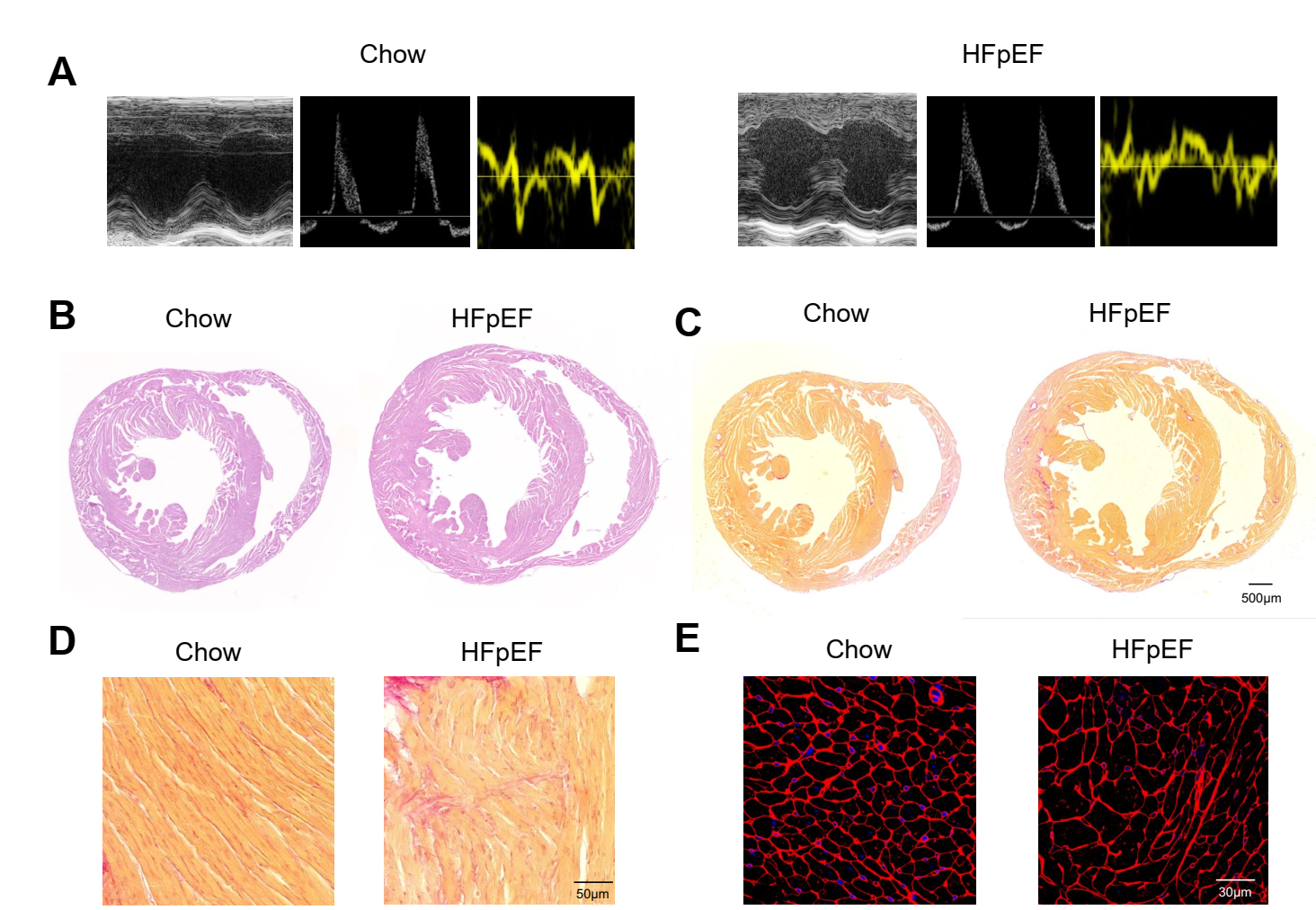


Figure S1. The representative images of pathological and echocardiographic results of the “two-hit” HFpEF model. A, Representative images of echocardiographic results of the chow and HFpEF groups. B,C) The H&E staining and Sirius red staining (low power field) of the chow and HFpEF groups. D) The Sirius red staining (high power field) of the chow and HFpEF groups. E) WGA staining of the chow and HFpEF groups.


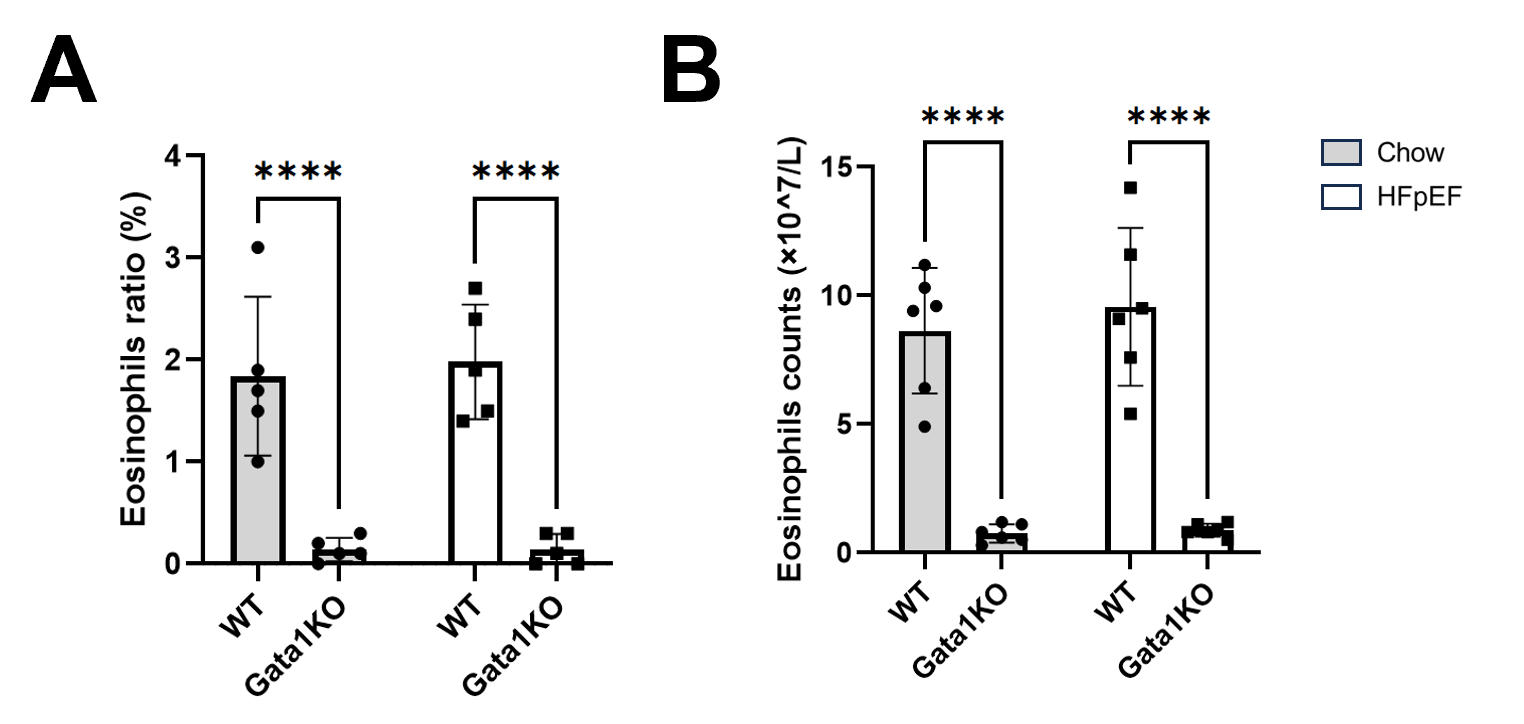


Figure S2. The ratio (A) and absolute counts (B) of blood eosinophil counts. n=6 per group. **** *p*<0.0001.
